# Hydrogen-rich saline attenuates spinal cord hemisection-induced testicular injury in rats

**DOI:** 10.18632/oncotarget.15876

**Published:** 2017-03-03

**Authors:** Li Ge, Li-Hua Wei, Chang-Qing Du, Guo-Hua Song, Ya-Zhuo Xue, Hao-Shen Shi, Ming Yang, Xin-Xin Yin, Run-Ting Li, Xue-er Wang, Zhen Wang, Wen-Gang Song

**Affiliations:** ^1^ Department of Histology and Embryology, Taishan Medical University, Tai-an City, PR China; ^2^ Key Laboratory of Atherosclerosis in Universities of Shandong, Taishan Medical University, Institute of Atherosclerosis, Taishan Medical University, Tai-an City, PR China; ^3^ Department of Basic Nursing Teaching, Taishan Medical University, Tai-an City, PR China; ^4^ Department of Clinical Medicine, Taishan Medical University, Tai-an City, PR China; ^5^ Department of Physiology, Shandong University School of Medicine, Jinan, Shandong, PR China; ^6^ Department of Medical Immunology, Taishan Medical University, Tai-an City, PR China

**Keywords:** hydrogen-rich saline, hemisectioned spinal cord injury, rat, testis, spermatogenic cell

## Abstract

To study how hydrogen-rich saline (HS) promotes the recovery of testicular biological function in a hemi-sectioned spinal cord injury (hSCI) rat model, a right hemisection was performed at the T11–T12 of the spinal cord in Wistar rats. Animals were divided into four groups: normal group; vehicle group: sham-operated rats administered saline; hSCI group: subjected to hSCI and administered saline; HRST group: subjected to hSCI and administered HS. Hind limb neurological function, testis index, testicular morphology, mean seminiferous tubular diameter (MSTD) and seminiferous epithelial thickness (MSET), the expression of heme oxygenase-1 (HO-1), mitofusin-2 (MFN-2), and high-mobility group box 1 (HMGB-1), cell ultrastructure, and apoptosis of spermatogenic cells were studied. The results indicated that hSCI significantly decreased the hind limb neurological function, testis index, MSTD, and MSET, and induced severe testicular morphological injury. The MFN-2 level was decreased, and HO-1 and HMGB-1 were overexpressed in testicular tissues. In addition, hSCI accelerated the apoptosis of spermatogenic cells and the ultrastructural damage of cells in the hypophysis and testis. After HS administration, all these parameters were considerably improved, and the characteristics of hSCI testes were similar to those of normal control testes. Taken together, HS administration can promote the recovery of testicular biological function by anti-oxidative, anti-inflammatory, and anti-apoptotic action. More importantly, HS can inhibit the hSCI-induced ultrastructural changes in gonadotrophs, ameliorate the abnormal regulation of the hypothalamic-pituitary-testis axis, and thereby promote the recovery of testicular injury. HS administration also inhibited the hSCI-induced ultrastructural changes in testicular spermatogenic cells, Sertoli cells and interstitial cells.

## INTRODUCTION

Spinal cord injury (SCI) is defined as acute traumatic injury to the spinal cord, potentially leading to varying degrees of motor and/or sensory deficits, paralysis, and reduced quality of life [1, 2]. Despite great advances in the field of rehabilitation medicine, infertility due to SCI remains a significant complication. Fertility problems in men with SCI are mainly due to erectile or ejaculatory disturbances and poor semen quality [3, 4]. Advanced assisted reproductive technologies (ART) are often required to assist conception in couples with SCI male partners [5]. ART is selected on the basis of the number of motile spermatozoa available in the ejaculate. *In vitro* fertilization (IVF) and intracytoplasmic sperm injection (ICSI) are the only alternatives for men with severely reduced numbers of motile spermatozoa. Apart from the increased costs associated with IVF/ICSI, there are increased risk of complications to the mothers, multiple births, and possible mental retardation and autism in the offspring [6].

Some recent researchers have found that hydrogen gas (H_2_) has therapeutic effects by reduction of oxidative stress, inflammation, and apoptosis [7, 8]. In regard to the current research, inhalation of H_2_, drinking hydrogen-rich water(HRW) and injecting hydrogen-rich saline(HS) are the most common, convenient, safe and effective routes of H_2_ administration [9, 10]. Inhalation of H_2_ is a straightforward therapeutic approach. As early as 2001, Gharib et al. reported the anti-inflammatory effects of H_2_ on parasite-induced liver inflammation [11]. Recently, Watanabe et al. demonstrated that radiation-induced rat skin injury can potentially be alleviated by the pre-inhalation of hydrogen-containing gas [12]. Drinking HRW has been reported to have preventative or/and therapeutic effects in many stress-induced organ injuries [13, 14].

Even though oral administration is safe and convenient, H_2_ in water tends to escape over time and some H_2_ is lost in the stomach or intestine, making it difficult to control the concentration of H_2_ administered. Administration of H_2_ via an injectable HS vehicle may allow the delivery of more accurate concentrations of H_2_ [15]. HS is portable, easy to administer, and a safe mode of H_2_ delivery in the clinical setting. Intraperitoneal or intravenous HS injection to various animal models have been met with great success. HS administration was found to attenuate stress-induced gastric ulceration [16], acute carbon monoxide poisoning in rats [17], intestinal [18] and myocardial [19] ischemia-reperfusion injuries in rats, and radiation-induced immune dysfunction in mice [20]. In our previous study, we found that HS administration could reduce retinal excitotoxic injury and promote retinal recovery in glutamate-induced excitotoxic injury guinea pig model [21]. We also found that HS exerts neuroprotection against hypoxic-ischemic brain damage in neonatal mouse [22]. In addition, HS has potential therapeutic effects on contusive SCI [23, 24] and spinal cord ischemia-reperfusion injury [25]. However, to the best of our knowledge, no study has investigated the potential effects of HS on the recovery of testicular biological function, spermatogenesis, and etiology after SCI. Based on these facts, we hypothesized that HS is a potential therapeutic treatment to alleviate SCI-induced testicular injury. Therefore, this study, using a well-established model of hemisectioned SCI (hSCI) in rats [26], was designed to investigate whether HS administration could attenuate hSCI-induced testicular injury and to study the mechanisms underlying this effect.

## RESULTS

### Hind limb neurological function (BBB score)

**Figure 1:**
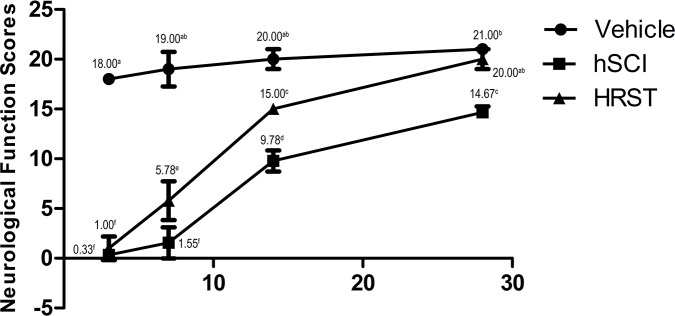
HS treatment improved the neurological function of the hind limbs of rats with hSCI Neurological function was assessed at 3, 7, 14, and 28 days after surgery. Data are expressed as means ± SD. (n = 9 per group). The Basso, Beattie, Bresnahan (BBB) locomotor scores demonstrated that spinal cord injury markedly affected the hind limb neurological functions of the rats (Figure 1). HS treatment improved the recovery of hind limb neurological function. The rats in the HRST group had the highest BBB scores 28 days after injury (20.00 ± 1.00).

To observe the protective role of HS, we investigated the effects of different dose durations (3, 7, 14, and 28 days) of HS on the hind limb neurological function scores of hSCI animals (Figure 1). All the animals survived until the final neurological behavior assessment at 28 d after hSCI. The results indicated that sham operation did not seriously injure rat hind limb neurological function compared with the control group (the BBB score of all animals in the control group was 21). The BBB score indicated that hSCI caused significant deficit in the hind limb neurological function, which was markedly improved by HS treatment at 3, 7, 14 and 28 days after hSCI (*p* < 0.05 vs. the hSCI group, n = 9 per group, Figure 1). In particular, the hind limb neurological function recovered more quickly in the HRST group after 7 and 14 days of the surgery. The animals in the HRST group exhibited the highest BBB score 28 days after injury (20.00 ± 1.00), there is no significant difference compared with the control group. The animal's hind limb neurological function in the hSCI group improved over time, but the recovery speed was significantly slower in this group relative to the HRST group throughout the duration of the experiment. The BBB score of the hSCI group remained significantly lower than that of the HRST group (14.67 ± 0.58^c^ vs. 20.00 ± 1.00^ab^, *p*<0.01).

### Effects of HS administration on the testis index

**Figure 2:**
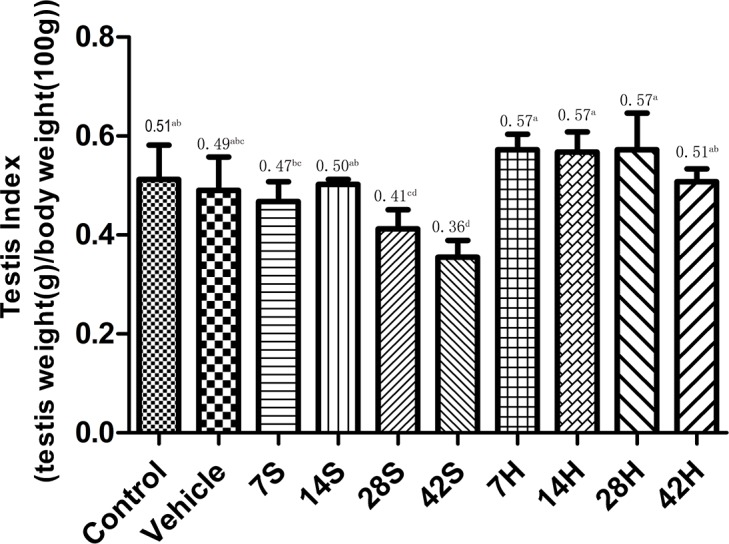
Effects of HS treatment on the testis index after hSCI The 7S, 14S, 28S, and 42S subgroups were the subgroups analyzed 7, 14, 28, and 42 days after hSCI, respectively. In the HRST group, the 7H, 14H, 28H, and 42H subgroups indicated 7, 14, 28, and 42 days of HS treatment after hSCI. The testis indices of rats were significantly decreased in the hSCI group than in the control and vehicle groups (0.41 ± 0.04^cd^, 0.36 ± 0.03^d^ vs. 0.51 ± 0.03^ab^, 0.49 ± 0.04^abc^). The testis indices were significantly higher in the HRST group than in the hSCI group (0.51 ± 0.03^ab^ vs. 0.36 ± 0.03^d^). Values with different superscript letters (a–d) above the bars differ significantly (*p* < 0.05). Data are expressed as means ± SD. (n = 6 per group).

The testis indices are shown in Figure 2. hSCI resulted in a significant decrease in the testis index compared with the control group, particularly at 28 and 42 days after injury. Meanwhile, the testis index reduced to a significantly lesser extent in the HRST group (*p* < 0.05) than in the hSCI group (Figure 2). There was no significant difference in the testis indices between the control and vehicle groups.

### Effects of HS administration on testis morphology (MSTD and MSET)

**Figure 3:**
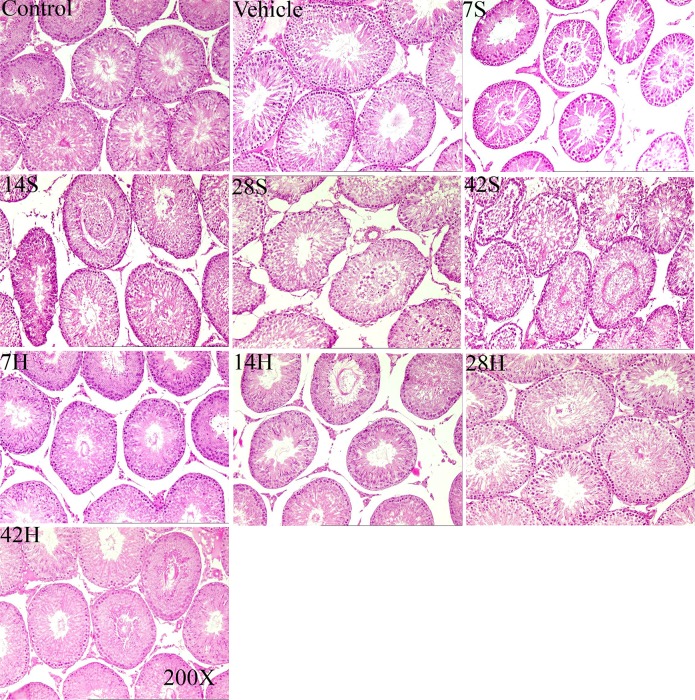
Representative photomicrographs of testicular H&E stained slices in the control group (control), vehicle group(vehicle), hSCI groups(7S, 14S, 28S, 42S) and HRST groups (7, 14, 28, and 42 h) Testicular tissues in the control and vehicle groups demonstrated regular morphology of seminiferous tubules with an orderly arrangement of spermatogenic cells. The integrity of the tubules was lightly impaired in the 7S and 14S subgroups, the layers of seminiferous epithelium were decreased, and deciduous spermatogenic cells could be seen in the lumen. In the 28S and 42S subgroups, the seminiferous tubules showed significant shrinkage, the structure of seminiferous epithelium loosened, and the seminiferous epithelial cells were more disorderly. The histological appearance of testicular tissue in the HRST groups was improved.

The testicular morphology was observed by staining slices of testicular tissue with H&E. Figure 3 shows the histopathological findings in all the groups. In the control and vehicle groups, testicular tissues demonstrated a regular morphology of seminiferous tubules with an orderly arrangement of spermatogenic cells including spermatogonia, primary spermatocytes, secondary spermatocytes, spermatids, and spermatozoa (Figure 3).

However, in the hSCI group, prominent morphological changes of seminiferous tubules appeared at 1 and 2 weeks after hSCI. The integrity of the tubules was lightly impaired. The layers of seminiferous epithelium was decreased, and the numbers of spermatids and spermatozoa were decreased and arranged in a disorderly fashion in some tubules. Tubules were observed along with the presence of deciduous spermatogenic cells in the lumen (Figure 3). At 4 weeks after surgery, seminiferous tubules appeared more severely injured: the tubules showed hypospermatogenesis, maturation arrest with loss of germ cells as indicated by the presence of deciduous germ cells in the lumen, and tubular shrinkage. At 6 weeks after injury, the morphological changes in the seminiferous tubules were more dramatic (Figure 3). The tubules showed significant shrinkage, the structure of the seminiferous epithelium loosened, and the seminiferous epithelial cells were more disorderly. The tubules with deciduous spermatogenic cells in the lumen could be easily seen. In the HRST subgroups, testicular tissue showed an improved histological appearance relative to the hSCI group, and the morphological changes were ameliorated. Many seminiferous tubules appeared normal, with an almost regular arrangement of spermatogenic cell layers, and some contained viable spermatozoa (Figure 3).

**Figure 4:**
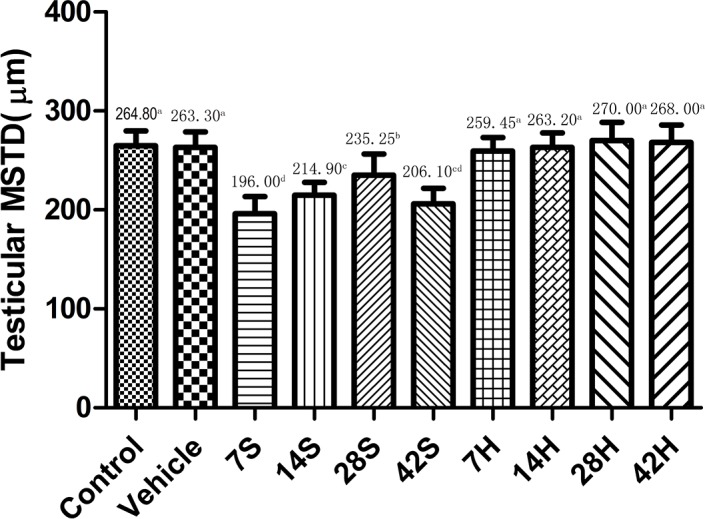
Effects of HS administration on testicular MSTD after hSCI The testicular MSTD was significantly decreased in the hSCI group than in the control and vehicle groups (206.10 ± 15.83^cd^ vs. 264.80 ± 15.18^a^ and 263.30 ± 15.43^a^). However, the MSTD was significantly higher in the HRST group than in the hSCI group (268.00 ± 17.75^a^ vs. 206.10 ± 15.83^cd^). Values with different superscript letters (a–d) above the bars differ significantly (*p* < 0.05). Data are expressed as means ± SD.

**Figure 5:**
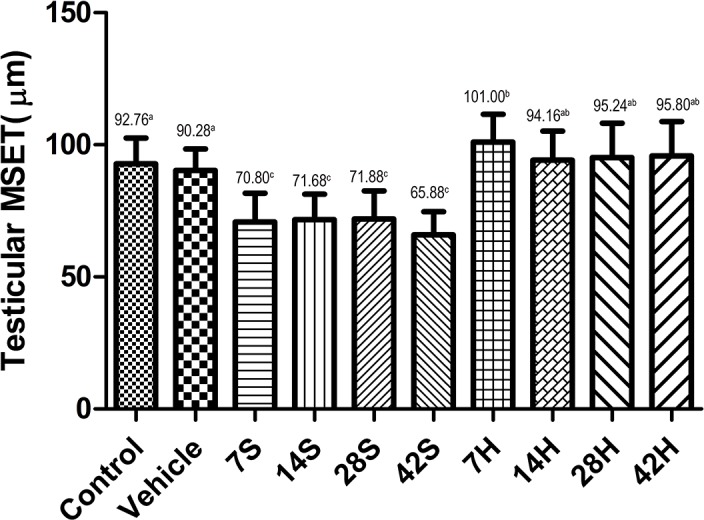
Effects of HS treatment on testicular MSET after hSCI The testicular MSET was significantly decreased in the hSCI group than in the control and vehicle groups (65.88 ± 5.83^c^ vs. 92.76 ± 8.85^a^ and 90.28 ± 8.22^a^). Note that HRST groups showed a significant increase in MSET (95.80 ± 7.04^ab^ vs. 65.88 ± 5.83^c^). Values with different superscript letters (a–c) above the bars differ significantly (*p* < 0.05). Data are expressed as means ± SD.

As shown in Figures 4 and 5, the MSTD and MSET in the hSCI group were markedly decreased compared to those in the control and vehicle groups after injury. In contrast, the MSTD and MSET were significantly higher in the HRST group than in the hSCI group (*p* < 0.05).

### Effects of HS administration on HO-1 expression

**Figure 6:**
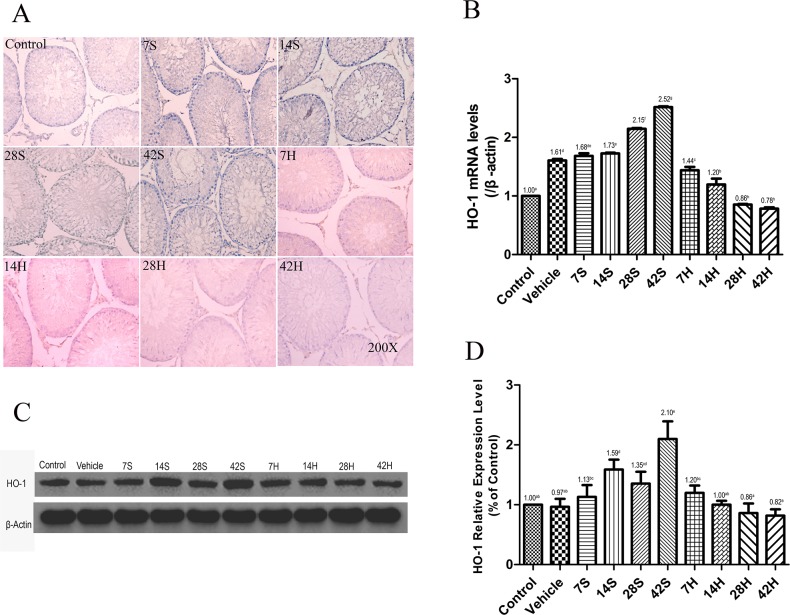
HS treatment inhibited the expression of HO-1 **(A)** Representative photomicrographs of immunohistochemical staining of HO-1. **(B)** Relative expression level of HO-1 mRNA in different groups. β-Actin was used as the internal control gene. Real-time qRT-PCR revealed an increase in the HO-1 mRNA level at 42 days after hSCI in the hSCI group than in the control and vehicle groups (2.52 ± 0.01^g^ vs. 1.00±0.00^a^ and 1.61 ± 0.03^d^), whereas HS administration significantly inhibited HO-1 mRNA overexpression (0.78 ± 0.02^h^ vs. 1.00 ± 0.00^a^ and 1.61 ± 0.03^d^). **(C)** Western blot analysis of HO-1 protein expression levels in different groups. β-Actin was used as a loading control and was detected with anti-β-actin antibodies. **(D)** Bands were analyzed using the Image-Pro-Plus System. Each bar corresponds to the mean ± SD. for 3 independent experiments. Western blot analysis revealed reduced HO-1 expression in the HRST group than in the hSCI groups (0.82 ± 0.11^a^ vs. 2.10 ± 0.19^e^). Values with different superscript letters (a–h) above the bars differ significantly (*p* < 0.05).

As shown in Figure 6A, the areas stained positive for HO-1 were stained brownish, and the control group showed weakly positive for HO-1. Marked activation of HO-1 was observed in the majority of germ cells after hSCI, especially near the luminal surface of seminiferous tubules. In contrast, HO-1 expression was obviously decreased in the HRST group than in the hSCI group (Figure 6A). Similarly, the results of the real-time qRT-PCR and Western blot analysis revealed that the HO-1 mRNA transcript and protein levels were considerably higher in testicular tissue of the hSCI group, while HO-1 expression was reduced in the HRST group (Figure 6B, 6C, and 6D).

### Effect of HS administration on MFN-2 expression

**Figure 7:**
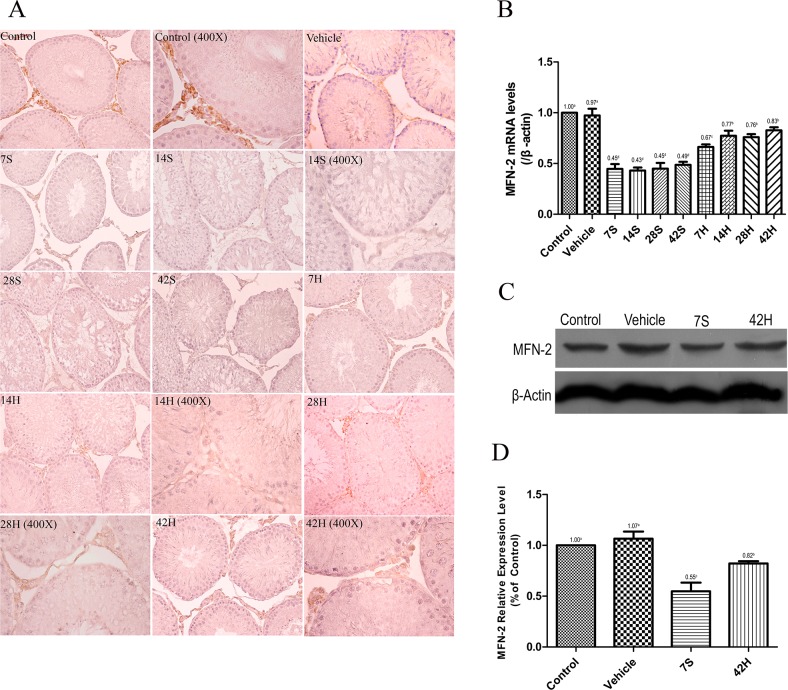
HS administration increased the expression of MFN-2 **(A)** Representative photomicrographs of the immunohistochemical staining of MFN-2. Photomicrographs marked with 400× indicate that the magnification was 400×; the remaining photomicrographs were taken at a magnification of 200×. **(B)** The relative expressional level of MFN-2 mRNA in different groups. Real-time qRT-PCR analysis showed that lower MFN-2 mRNA transcript levels in the hSCI group than in the control and vehicle groups (0.43 ± 0.03^d^ vs. 1.00 ± 0.00^a^, 0.97 ± 0.07^a^). The MFN-2 mRNA expression was significantly higher in the HRST group than in the hSCI group (0.83 ± 0.03^b^ vs. 0.43 ± 0.03^d^). In contrast, the expression of MFN-2 mRNA was still lower in the HRST group than in the control and vehicle groups. **(C)** Western blot analysis of the MFN-2 expression levels. β-Actin was used as the loading control and detected with anti-β-actin antibodies. **(D)** Bands were analyzed using the Image-Pro-Plus System. Each bar corresponds to the mean ± SD. for three independent experiments. Western blot analysis indicated a dramatic increase in the MFN-2 protein expression in the HRST group than in the hSCI group; however, the MFN-2 expression was still lower in the HRST group than in the control and vehicle groups. Values with different superscript letters (a–d) above the bars differ significantly (*p* < 0.05).

MFN-2 was primarily expressed in the testicular interstitial cells of the control and vehicle groups (Figure 7A). Interstitial cells were stained brownish-yellow in each group. The MFN-2 level was significantly reduced 7 days after injury, while MFN-2 in the cytoplasm was markedly activated in the HRST group, especially at 14 days after HS administration (Figure 7A). Real-time qRT-PCR and Western blot analysis indicated that the transcriptional level and immunoreactivity of MFN-2 were greatly decreased in the testicular tissues in the hSCI group, but MFN-2 expression was significantly increased in the HRST group (Figure 7B, 7C, and 7D). These findings were consistent with the immunohistochemistry results.

### Effect of HS administration on HMGB-1 expression

**Figure 8:**
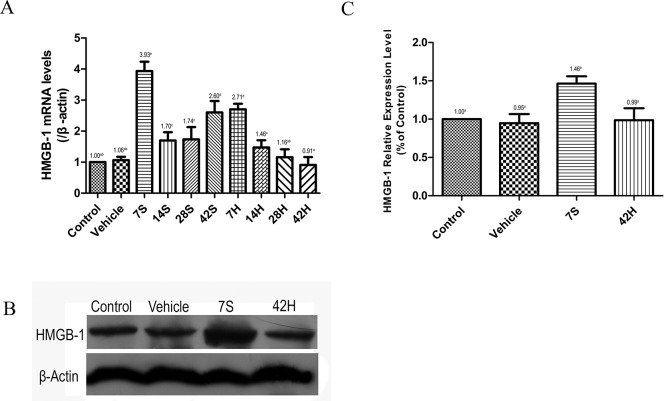
HS injection inhibited the expression of HMGB-1 **(A)** Relative expressional level of HMGB-1 mRNA in different groups. Real-time qRT-PCR analysis showed that HMGB-1 mRNA expression significantly increased after hSCI, however HS injection inhibited the expression of HMGB-1 mRNA. **(B)** Western blot analysis. Western blot analysis of the expression level of HMGB-1 in the four treatment groups. β-Actin was used as the loading control and detected with anti-β-actin antibodies. **(C)** Bands were analyzed using the Image-Pro-Plus System. Each bar corresponds to the mean ±SD. for 3 independent experiments. Western blot analysis indicated that HS injection inhibited the expression of HMGB-1. There is no significant difference in the expressional level of HMGB-1 among control, vehicle and HRST group. Values with different superscript letters (a–d) above the bars differ significantly (*p* < 0.05).

We performed real-time qRT-PCR and Western blot analysis to exmine the expression of HMGB-1 (Figure 8). The HMGB-1 mRNA and protein levels were markedly increased at 7 days after the injury in the hSCI group, while the levels in the HRST group (42H subgroup) were similar to those in the vehicle and control group.

### Effect of HS administration on the ultrastructures of the hypophysis and testis

**Figure 9:**
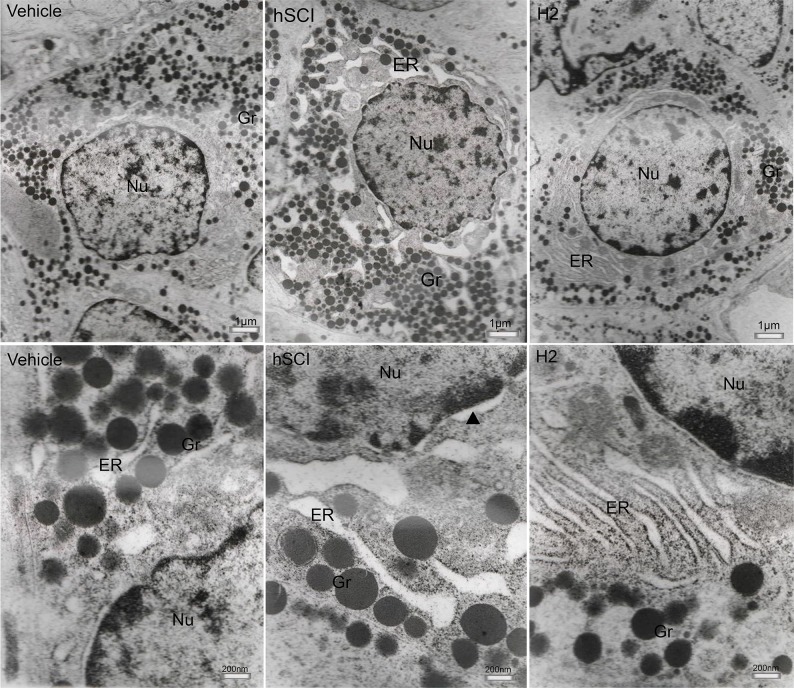
Ultrastructural changes in the gonadotroph cells of pituitary Representative TEM micrographs showing the ultrastructure of gonadotroph cells. (Upper three microphotographs were taken low magnification and the lower three were taken at high magnification.) hSCI: hSCI group; H2: HRST group; Nu: Nucleus; ER: Endoplasmic reticulum; Gr: Secretory granules; ▲: perinuclear space, the same as follows. Less damage of gonadotroph cells was observed in vehicle group, including dilation of the ER, the slight damage of nuclear and mitochondrial membrane. After hSCI, severe damage was observed in hSCI group, including cellular swelling, dilation of perinuclear spaces and ER. There was only slight changes in HRST group.

Results of the TEM revealed that in the vehicle group, except for dilation of the endoplasmic reticulum (ER), slight damage to the nuclear and mitochondrial membrane in a few gonadotrophs (Figure 9), the majority of cells had good ultrastructures, nuclear membrane, and ER pool. In contrast, gonadotrophs in the hSCI group exhibited serious damage and swelling, crenulated nuclei, and dilated perinuclear spaces. In addition, ER pools were universally dilated in this group. Interestingly, gonadotrophs in the HRST group exhibited nearly normal ultrastructures, intact nuclear membrane and ER pool, with no enlargement in the ER pools.

**Figure 10:**
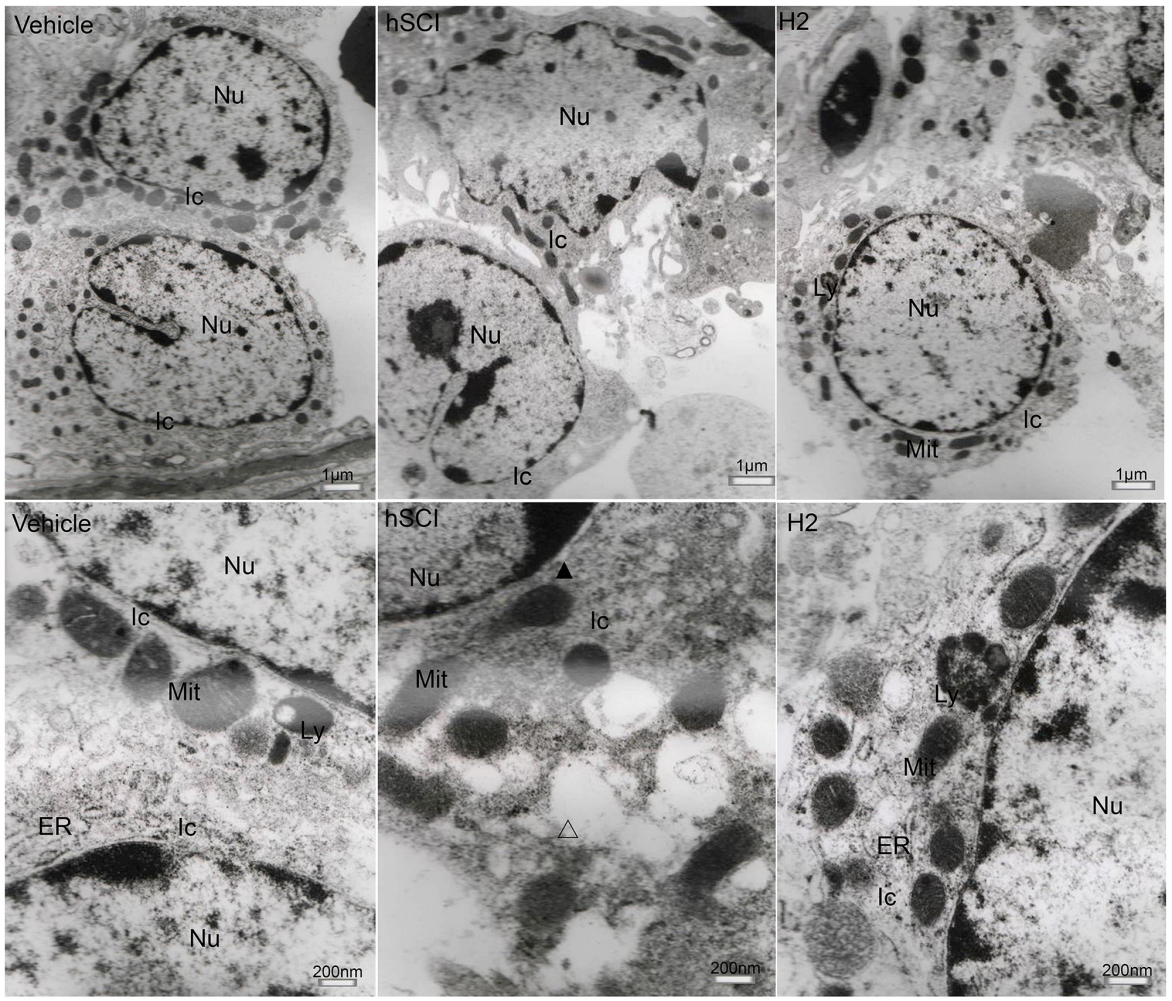
Ultrastructural changes of testicular interstitial cells Representative TEM micrographs showing the ultrastructure of testicular interstitial cells. (Upper three microphotographs were taken low magnification and the lower three were taken at high magnification.) Ic: interstitial cells; Ly: lysosomes; Mit: mitochondria; Δ: vacuoles in the cytoplasm of interstitial cells. Testicular interstitial cells in the hSCI group exhibited severe damage, including cellular swell, dilation of perinuclear spaces (▲), discontinuous mitochondrial outer membranes, fuzzy mitochondrial cristae, and cytoplasmic vacuolization (Δ). No prominent alterations were observed in the HRST group when compared with the vehicle group.

**Figure 11:**
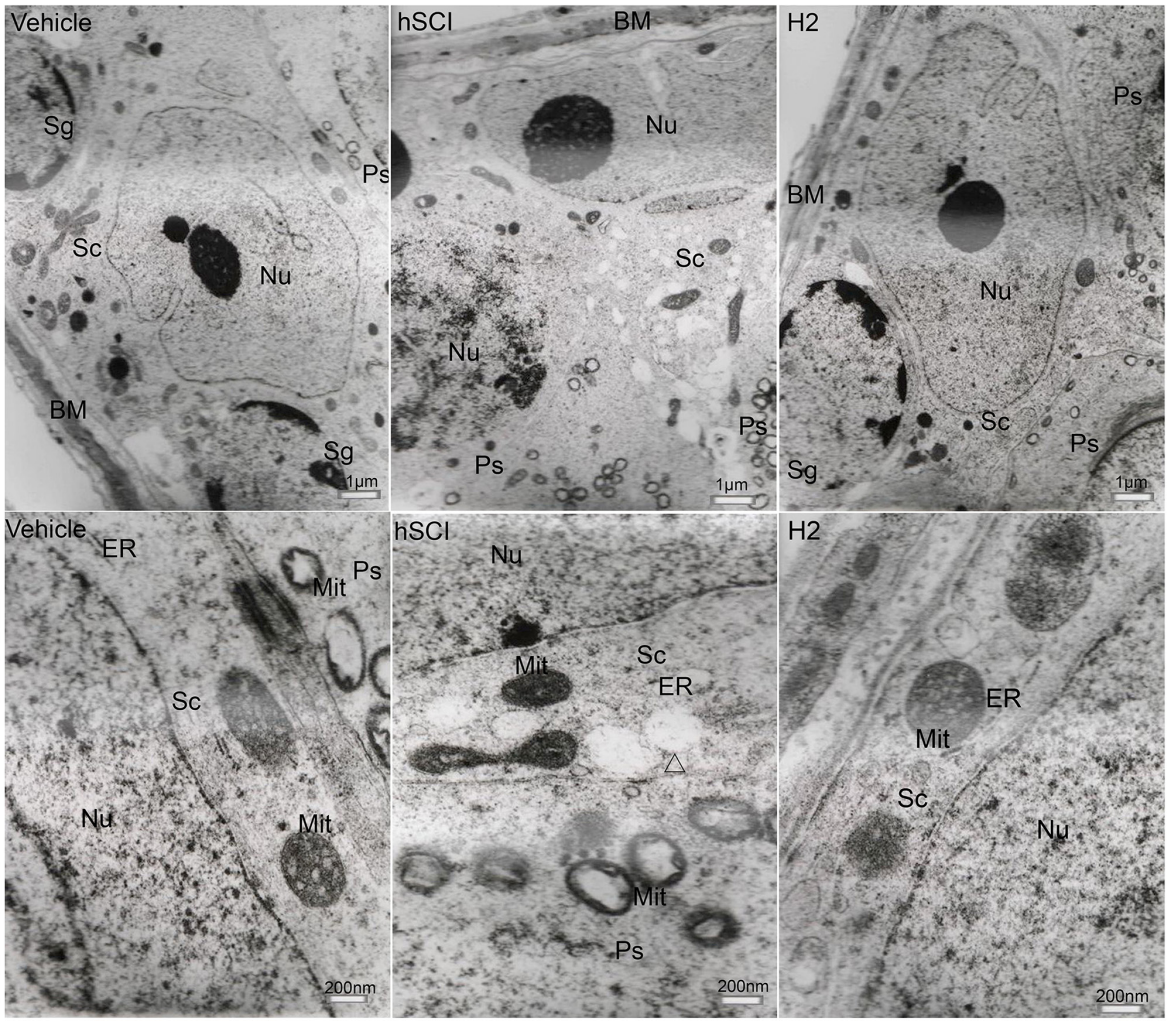
Ultrastructural changes in spermatogenic cells and testicular Sertoli cells Representative TEM microphotographs showing the ultrastructure of spermatogenic cells and Sertoli cells. (The upper three microphotographs were taken at low magnification and the lower three were taken at high magnification.) Sg: Spermatogonia; Sc: Sertoli cells; Ps: Primary spermatocytes; BM: Basal membrane; Δ: vacuoles in the cytoplasm of Sertoli cells. Sertoli cells in the hSCI group exhibited severe damage, including cytoplasmic vacuolization, dilation of ER pools, and discontinuous mitochondrial outer membranes. Spermatogonia in this group appeared swollen. No significant alterations were observed in the HRST group when compared with the vehicle group.

**Figure 12:**
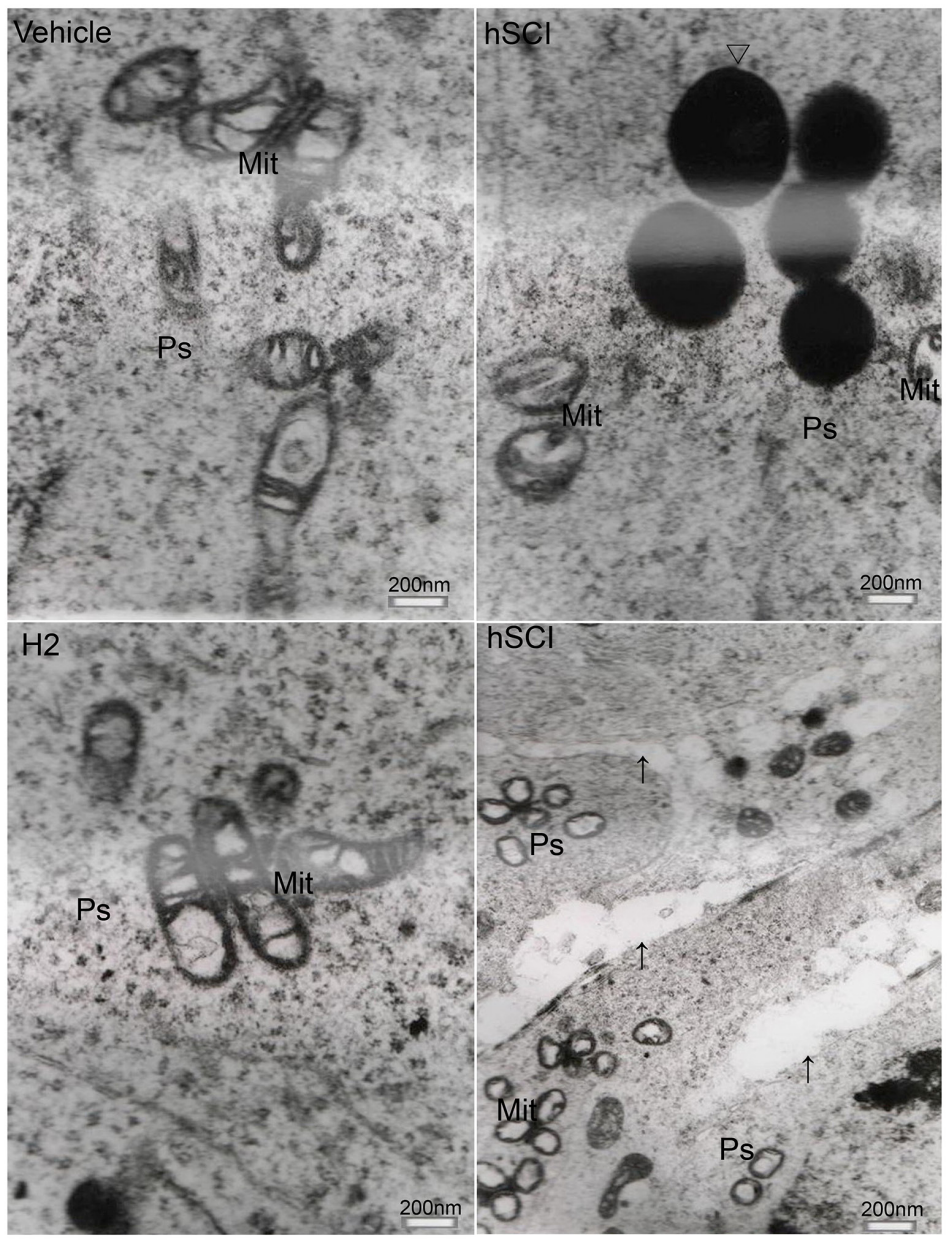
Ultrastructural changes of primary spermatocytes ↑: Space between primary spermatocytes; Δ: Pathological protein body Representative TEM microphotographs showing the ultrastructure of primary spermatocytes. Severe damage of primary spermatocytes was observed in the hSCI group, including enlargement of cellular spaces, mitochondrial vacuolization, reduced or absent mitochondrial cristae, appearance of pathological protein bodies in the cytoplasm. No significant alterations were observed in the HRST group compared to the vehicle group.

The ultrastructure of interstitial cells in the hSCI group exhibited varying degrees of injury (Figure 10). Sertoli cells in the hSCI group also showed significant damage, with swollen vacuoles in the cytoplasm (Figure 11). ER pools were dilated, and the mitochondrial outer membranes were discontinuous. TEM revealed that the spermatogonia located around Sertoli cells were also swollen, and there were fewer spermatogonia compared to the other groups. The spaces between the primary spermatocytes were broadened (Figure 12). Most of the mitochondria in the cytoplasm of primary spermatocytes were vacuolized, and the number of mitochondrial cristae was reduced, while some cristae disappeared (Figure 12). Surprisingly, pathological protein bodies appeared in the cytoplasm of primary spermatocytes in the hSCI group alone. No significant alterations were observed in the HRST group relative to the vehicle group (Figure 11).

### Effect of HS administration on germ cell apoptosis

**Figure 13:**
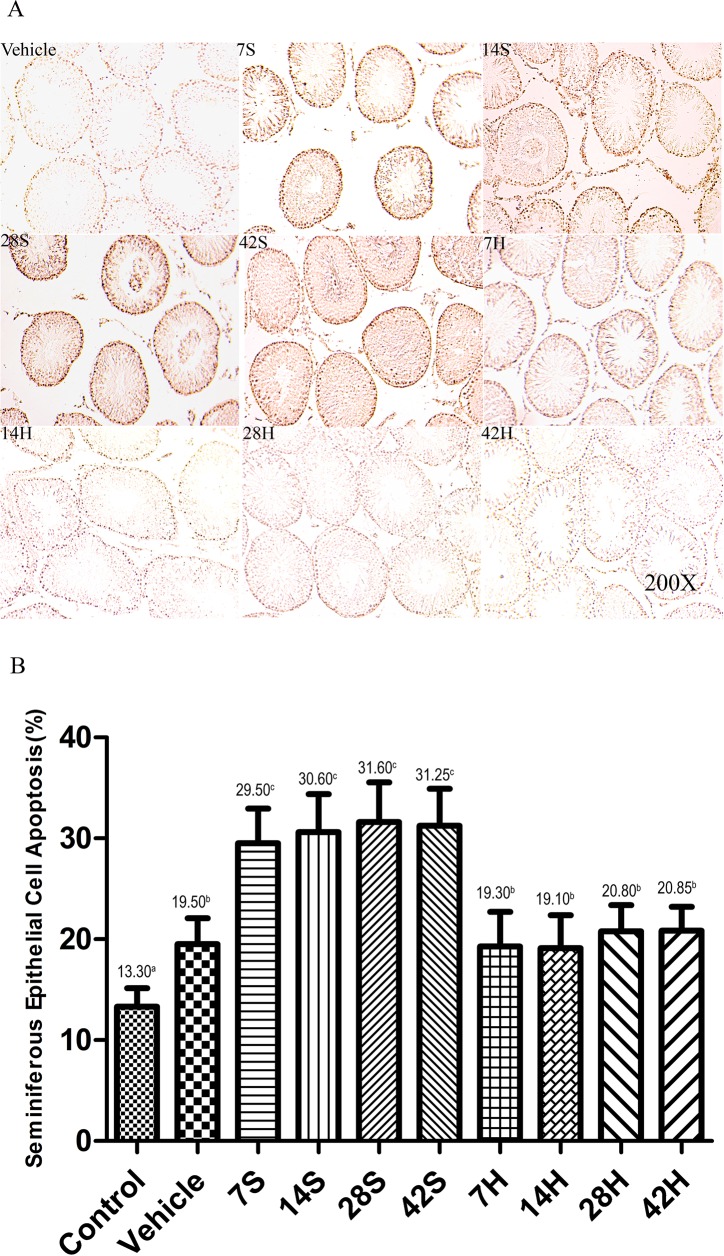
HS administration prevents the hSCI-induced testicular germ cell apoptosis Representative micrographs of TUNEL staining of seminiferous tubules in the vehicle, hSCI (7S, 14S, 28S, and 42S), and HRST (7H, 14H, 28H, and 42H) groups. Apoptotic cells in the seminiferous tubules were identified as those with a brown-stained nucleus. Apoptotic cells were observed mainly in spermatogonia and spermatocytes. Magnification: 200×. Quantitative analysis of apoptotic cells in different groups. hSCI markedly increased the number of TUNEL-positive cells when compared with the control and vehicle groups (31.25 ± 2.65^c^ vs. 13.30 ± 1.04^a^ and 19.50 ± 1.58^b^, respectively). Note that the number of TUNEL-positive cells in the HRST group showed a significant decrease when compared with that of hSCI group (20.85 ± 1.37^b^ vs. 31.25 ± 2.65^c^). Values with different superscript letters (a–c) differ significantly (*p* < 0.05). Data are expressed as means ± SD. (n = 6 per group).

As shown in Figure 13A, cells in the seminiferous epithelium whose nuclei stained brown were identified as apoptotic. Based on their position in the tubules, they were primarily identified as spermatogonia and spermatocytes. The results of the TUNEL assay confirmed the histopathological findings. There were fewer TUNEL-positive cells in the testicular tissue of the vehicle group than in that of the hSCI group (Figure 13B). The mean number of TUNEL-positive cells per seminiferous tubule was significantly higher in the hSCI group than in the vehicle and control groups (31.25 ± 2.65^c^ vs. 19.50 ± 1.58^b^ and 13.30 ± 1.04^a^, respectively; *P* < 0.05). Furthermore, HS had an obvious therapeutic effect on damaged testis, as evidenced by the significant reduction in the number of TUNEL-positive cells in the HRST group relative to the hSCI group (20.85 ± 1.37^b^ vs. 31.25 ± 2.65^c^, respectively; *P* < 0.05).

## DISCUSSION

In recent years, an increasing number of reports have investigated the preventative and therapeutic effects of H_2_ in many diseases and organs, such as stress-induced gastric ulceration [16], acute carbon monoxide poisoning in rats [17], intestinal [18] and myocardial [19] ischemia-reperfusion injuries in rats, and radiation-induced immune dysfunction in mice [20]. However, there is no *in vivo* evidence that HS injection is an effective approach for the treatment of hSCI-induced decreased testicular function. Our results demonstrated that intraperitoneal injection of HS can significantly improve the hind limb motor function, increase the testis index, MSTD, and MSET. In aspect of morphology, HS administration could alleviate abnormal structure and arrangement of spermatogenetic cells induced by hSCI, suggesting HS plays protective effects in hSCI development.

HO-1 is a member of the heat shock protein family and is highly inducible by a vast array of stimuli [30]. Many studies have shown that HO-1, when induced, plays a significant protective role against inflammatory processes and oxidative tissue injury [31, 32]. The protective effect of HO-1 on brain damage has been shown to have the highest threshold. HO-1 mitigates oxidative injury when the expression level of HO-1 was upregulated by less 5× than baseline; however, it may have oxidative toxicity when upregulated by more than 15× [33]. Here, we examined the expression of HO-1 to investigate the stress response of testicular tissue to hSCI. Previous research has shown that HO-1 was mainly expressed in testicular Sertoli cells [28]. We found that HO-1 expression levels increased persistently with time after the SCI injury. We speculated that HO-1 may contribute to alleviating hSCI-induced oxidative stress of testicular tissue in the early stages of injury. However, HO-1 overexpression over time may result in oxidative toxicity. H_2_ may alleviate hSCI-induced oxidative toxicity in testicular tissue of rats via the HO-1/CO system, which is a well-known signaling system confirmed in animals and recently in plants [34].

Recent studies have shown that MFN-2 plays a strong role in the occurrence and development of various diseases [35]. We found that MFN-2 was expressed mainly in the interstitial cells of the testes in the control and vehicle groups. MFN-2 expression was significantly decreased after hSCI, and HS injection obviously inhibited the decrease of the MFN-2 concentration in the testicular tissues. Furthermore, the ultrastructure of interstitial cells was found to have varying degrees of injury, in particular, the mitochondrial outer membranes were incomplete and cristae were indistinct following hSCI. It is encouraging that the ultrastructure of interstitial cells was basically normal in the HRST group. Therefore, we speculate that HS administration may alleviate the hSCI-induced ultrastructural injury of interstitial cells by inhibiting the decrease of MFN-2 expression.

HMGB-1, which is secreted by activated macrophages, neutrophils, and endothelial cells and released passively from necrotic cells, acts as an endogenous danger signal and inflammatory mediator [36, 37]. Therefore, inhibiting its release is important in anti-inflammatory therapy. HS has been reported to attenuate massive hepatectomy-induced liver injury not only by attenuating oxidative damage but also by reducing the production of inflammatory cytokines such as TNF-α, IL-6, and HMGB-1 [38]. Here, at 7 days after hSCI, HS administration obviously inhibited the elevation of the HMGB-1 level in the testicular tissues, suggesting that the protective effect of HS in the testicular tissue may be mediated by the suppression of the excessive testicular inflammatory response and its cascade induced by hSCI.

Previous research has demonstrated that the endocrine profiles of men with SCI are different from those of normal men: there is a high prevalence of hypothalamic-pituitary-testis axis abnormalities in the former. Furthermore, sperm motility and the percentage of normal sperm morphology were lower in SCI men than in normal control participants [39]. In the present study, we found severe ultrastructural damage in the gonadotrophs in the pituitary of hSCI rats. Furthermore, our findings revealed varying degrees of ultrastructural damage to testicular interstitial cells, Sertoli cells, and spermatogenic cells in the hSCI group. The pituitary has been shown to secrete luteinizing hormone and follicle-stimulating hormone, which in turn binds to the receptors on testicular interstitial cells and Sertoli cells, subsequently stimulating the production of testosterone and estrogens by interstitial cells and the production of estrogens, inhibin, and activin by Sertoli cells [40]. Therefore, we speculate that the ultrastructural damage of gonadotroph cells in the hSCI group most likely leads to hyposecretion of gonadotropin, triggering the dysfunction of interstitial cells and Sertoli cells, resulting in abnormal spermatogenesis. By contrast, HS administration could inhibit the hSCI-induced ultrastructural changes of gonadotroph cells, improve the recovery of testicular somatic cell function, and restore normal spermatogenesis.

Recently, it has been shown that a local “apical ectoplasmic specialization–blood–testis barrier–hemidesmosome/ basement membrane” functional axis (ES-BTB-hemidesmosome axis) can regulate the events of spermiation and BTB restructuring, via paracrine/autocrine factors and polarity proteins produced locally in the seminiferous epithelium [41]. ES is an atypical adherens junction uniquely found in the testis at the interface between Sertoli cells and elongating spermatids during spermiogenesis, known as apical ES [42]. The ES-BTB-hemidesmosome axis has been shown to regulate spermatogenesis [41]. Furthermore, other studies have indicated that SCI could cause sustained disruption of the BTB in rats by increasing BTB permeability, decreasing the expression of the tight junction protein occludin, and inducing testis immune cell infiltration and extensive germ cell apoptosis [43]. In the study, we speculate that ultrastructural abnormalities in the Sertoli cells in the hSCI group could contribute to the dysfunction of this ES-BTB-hemidesmosome axis and disruption of the BTB integrity, thus disturbing spermatogenesis. HS administration can inhibit the ultrastructural changes of Sertoli cells induced by hSCI, improve the recovery of ES-BTB-hemidesmosome axis function, BTB restructuring, and spermatogenesis.

It has been shown that HS administration could effectively protect testis against injury by alleviating germ cell apoptosis [44, 45]. In this study, extensive spermatogenic cell apoptosis was observed at 7 days after hSCI. Intriguingly, HS administration had an obviously therapeutic effect on hSCI-damaged testis, as evidenced by a significant reduction in the number of TUNEL-positive cells compared to the hSCI group. This result indicated that HS administration may also protect against hSCI-induced damage in the testis through the anti-apoptotic pathway.

In conclusion, we found that HS may reduce the hSCI-induced damage to the structure and function of the testis. The mechanism by which recovery of testis function occurs may involve recovery of hind limb motor function; increase of the testis index, MSTD, and MSET; inhibition of oxidative stress, inflammatory response and germ cell apoptosis; protection of the morphology and function of germ cells, Sertoli cells, testicular interstitial cells, and hypophysial gonadotrophs; alleviation of the functional abnormalities of hypothalamic-pituitary-testis axis and ES-BTB-hemidesmosome axis. Our findings suggest that intraperitoneal injection of HS can be a new breakthrough for the clinical treatment of SCI-induced male sterility.

## MATERIALS AND METHODS

Unless otherwise indicated, all chemicals were purchased from Sigma Chemical Co. (St. Louis).

### Hydrogen-rich saline (HS) production

HS was a gift from the Institute of Atherosclerosis, Taishan Medical University. H_2_ was dissolved in physiological saline for 6 h under high pressure (0.4 MPa) to a supersaturated level using HS-producing apparatus. The saturated HS was sterilized by gamma radiation and stored under atmospheric pressure at 4°C in an aluminum bag with no dead volume. HS was freshly prepared every week to maintain an H_2_ concentration of more than 0.6 mM. Gas chromatography was used to confirm the H_2_ content in the saline as described previously [27].

### Establishment of an hSCI rat model

A total of 90 healthy, sexually mature male Wistar rats weighing 260–320 g were purchased from the Experimental Animal Center of the Lukang Company, Shandong Province. The animal protocols were approved by the Animal Care and Use Committee of Taishan Medical University, Tai-an city, China. All animals were given free access to normal rat diet and water and maintained on a 12:12-h light/dark cycle (lights on at 06:00 h).

The animals were fed a standard diet for at least 1 week before the surgery, and then randomly divided into the following four groups: (1) normal control group (n = 9), which received no treatment; (2) vehicle group, in which rats were sham-operated and given intraperitoneal injection of 5–6 mL physiological saline per kg body weight once a day (n = 9), (3) hSCI group, in which rats were subjected to hSCI plus intraperitoneal injection of 5–6 mL/kg physiological saline (n= 36), (4) Hydrogen-rich saline-treated (HRST) group, in which rats were subjected to hSCI plus 5–6 mL/kg HS injection (n = 36). Of these, the hSCI and HRST groups were divided into four subgroups (n = 9 per subgroup), namely, 1, 2, 4, and 6 weeks after hSCI or HRST subgroups.

A standardized hSCI model described by Lang et al. [26] was used with revision. Briefly, the animals were anesthetized by intraperitoneal injection of pentobarbital sodium (30 mg/kg), and placed prone on an operating table covered by a warming blanket to maintain their body temperature at 37.0 ± 0.50°C. Laminectomy at the T10-T12 level was then performed. Fine forceps were used to remove the spinous process and lamina of the vertebrae, and a right hemisection was made at T11-T12. A fine scalpel was used to cut the spinal cord. The muscle and skin layers were then sutured. The HRST group received the intraperitoneal injection of 5mL/kg HS at 30 min after the surgery, once a day for 1, 2, 4 and 6 weeks in the 1-, 2-, 4-, and 6-week HRST subgroups. The vehicle and hSCI groups received an equal volume of physiological saline injection. The rats in the vehicle group received only a laminectomy. All the animals received prophylactic benzylpenicillin sodium antibiotic (intramuscular injection of 200000 IU once a day for 3 days after surgery) after the induction of injury. The study procedures were approved by the Animal Care and Use Committee of Taishan Medical University. The hSCI rat model was verified as follows: immediately after recovering from anesthesia, the rats exhibited loss of hind limb motor function on the injured side but retained feeling, while the contralateral hind limb lost feeling but could move smoothly.

### Hind limb neurological function test

Neurological function of the hind limb was evaluated at 3, 7, 14, and 28 days after surgery by using the Basso, Beattie, Bresnahan (BBB) scale in an open field [28, 29]. Each rat was placed in an open field and observed for 5 min. All behavioral assessments were done by two observers who were blinded to the subject's experimental treatment.

### Tissue sample collection

All 9 animals in each subgroup were sacrificed with high dose pentobarbital sodium (200 mg/kg) at specific time intervals after the surgery (1, 2, 4 and 6 weeks). Bilateral testes were removed and weighed, and the body weights were also determined.

### Testis index detection

Six randomly selected animals per group were used to obtain the mean values of bilateral testicular weight for the control and vehicle groups. The weights of the testes only on the injured side were measured in the hSCI and HRST groups. The testis index was determined as the ratio of the testis weight (g) to the body weight (per hectogram).

### Histopathological observation of the testis

The testes on the injured side were divided transversely into two halves. One half of the testes were placed in Bouin's fixative for histopathological evaluation, and the others were frozen in liquid nitrogen and stored at −80°C for biochemical analyses. For histopathological observation, the samples of testicular tissue were embedded in paraffin and sectioned into 5-μm slices. The slices were deparaffinised and rehydrated in xylene and graded ethanol to distilled water.

### Measurements of seminiferous tubular diameter (MSTD) and seminiferous epithelial thickness (MSET)

To study histopathological changes, all the slices were stained with hematoxylin and eosin (H&E). The diameter of seminiferous tubules and thickness of the seminiferous epithelium were evaluated in 20 seminiferous tubules from each slice by an ocular micrometer at 400× magnification, and cylindrical tubules were chosen as far as possible. The seminiferous tubule diameter of each testis was calculated according to the greatest dimension of the minor axis direction, and the mean value was taken as the MSTD. MSET was determined by measuring the thicknesses of the epithelium from the basement membrane to the free surface of the epithelium in the direction of the minor axis. The mean value was taken to be the MSET. The testicular tissues were examined by two observers who were blinded to the subject's experimental treatment.

### Testicular immunohistochemical staining

Testicular tissue was immunohistochemically stained to study the heme oxygenase-1 (HO-1) and mitofusin-2 (MFN-2) levels and the distribution of expression in the testes. Endogenous peroxidase activity was blocked in 3% H_2_O_2_ in distilled water (dH_2_O) for 30 min at room temperature, and then antigen retrieval was performed. Testicular slices were then blocked in phosphate-buffered saline (PBS) containing 1.5% normal goat serum and 1% bovine serum albumin (BSA) for 2 h at room temperature, and then incubated with the following primary antibodies overnight at 4°C: rabbit anti-HO-1 (1:100, Boshide, Wuhan, China) or anti-MFN-2 (1:200, Boshide, Wuhan, China) and then incubated with a non-biotin HRP immunohistochemical reagent from the kit for 30 min at room temperature. The slices were washed thrice in 0.01 M PBS (for 5 min each time) between each step, and the DAB reaction was carried out at room temperature to stain the proteins. The slices were counterstained with hematoxylin, dehydrated, hyalinized, and mounted with neutral gum. Negative controls were prepared by replacing the primary antibody with PBS. Three slices were randomly selected from each testis and five positive areas were chosen from each slice.

### Real-time quantitative reverse transcription polymerase chain reaction (real-time qRT-PCR)

Total RNA was extracted from frozen testis tissue using a standard TRIzol (Invitrogen, USA) RNA isolation method. Reverse transcription of RNA was carried out using an Easy Script First-Strand cDNA Synthesis Super Mix kit (Trans Gen Biotech, USA), according to the manufacturer's instructions. Specific primers designed for the amplification of HO-1, MFN-2, HMGB-1, and β-actin were verified by NCBI Blast; primer sequences are shown in Table 1. qRT-PCR was performed on a Rotor-Gene Q Quantitative Real-time PCR Amplifier (Qiagen, Germany) using SYBR Premix Ex Taq^TM^ (TaKaRa, Japan). PCR reactions were carried out in a total reaction volume of 20 μL using the following cycle conditions: one cycle at 95°C for 5 min followed by 30 cycles of 94°C for 30 s, 51.5°C for 45 s, and 72°C for 1 min. The gene expression levels in each sample were analyzed in duplicate and normalized to the expression level of β-actin. The results are expressed as relative gene expression using the ΔCt method.

**Table 1:** Primer sequences for Real-Time qRT-PCR

Gene	Forward primer (5′-3′)	Reverse primer (5′-3′)
β-actin	TGGAATCCTGTGGCATCCATG	ACGCAGCTCAGTAACAGTCCG
HO-1	AACCAGCGAGTGGAGCCTGCCG	ATGGTACAAGGAGGCCATCACC
MFN-2	AGCGTCCTCTCCCTCTGACA	TTCCACACCACTCCTCCGAC
HMGB-1	CAGTGTAATGAGAATGTGCCG	AGTGGACTTTGTGGACTAGTG

### Western blot analysis

Frozen testicular tissue was prepared with lysis buffer (150 mM NaCl, 20 mM Tris HCl [pH 7.4], 0.1% sodium dodecyl sulfate [SDS], 1.0% Nonidet P-40, 0.5% sodium-deoxycholate, 0.2 mM phenylmethylsulfonyl fluoride, protease inhibitor cocktails, and phosphates inhibitors). Equal amounts of proteins were separated by 10% SDS-polyacrylamide gel electrophoresis (PAGE), and electrotransferred to polyvinylidene fluoride (PVDF) membranes (Millipore, USA). The proteins were then blocked with 5% BSA for 2 h at room temperature. The membranes were incubated with appropriately diluted rabbit anti-rat primary antibodies for the detection of HO-1 (Abcam, UK), MFN-2 (Boshide, Wuhan, China), HMGB-1 (Santa Cruz Biotechnology Co., USA), and mouse anti-rat β-actin (Santa Cruz Biotechnology Co., USA) overnight at 4°C. The membranes were then incubated with the relevant secondary antibodies (HO-1, MFN-2, HMGB-1: anti-rabbit IgG, β-actin: anti-mouse IgG; all purchased from Boshide, Wuhan, China) for 2 h at 20°C. The proteins were detected with an enhanced chemiluminescence (ECL) detection system. β-Actin served as the internal control. The analysis was repeated thrice.

### Ultrastructure observation of the hypophysis and testis

To examine the protective effect of HS on the hypophysis and testicular tissue, 4 animals in each group were sacrificed by severing the carotid artery on day 7 after hSCI. Then, 3-5 pieces of hypophysis and testicular tissue (1 mm × 3 mm) were collected, fixed immediately in 3% glutaraldehyde (4°C) for 24 h, washed in 0.1 M PBS, further fixed in 1% osmium tetroxide for 3 h at room temperature, washed in 0.1 M PBS, dehydrated in an ascending alcohol series, soaked in propylene, embedded in Epon 812, sectioned on an LKB-V ultramicrotome, stained with uranyl acetate and lead citrate, and finally examined by transmission electron microscopy (TEM).

### Detection of germ cell apoptosis

Apoptosis of seminiferous epithelial cells was determined using terminal deoxynucleotidyl transferase-mediated nick end labeling (TUNEL) with an In Situ Cell Death Detection kit (Roche Co., Basel, Switzerland). Briefly, according to the manufacturer's protocols, testicular slices were dewaxed in xylene, rehydrated in a graded alcohol series, and placed in dH_2_O. The slices were then incubated in a 20 μg/ml proteinase K working solution for 15 min at room temperature. The slices were then covered with terminal deoxynucleotidyl transferase (TdT) enzyme and incubated for 1 h at room temperature. After incubation in converter-peroxidase-conjugated anti-fluorescein (POD) antibodies, the slices were stained with diaminobenzidine (DAB). Apoptotic cells in seminiferous tubules were identified as those with a brown-stained nucleus. The mean number of TUNEL-positive cells per 100 tubules were determined from three cross-sections from each rat under a light microscope at a magnification of 200×.

### Statistical analysis

There were at least six replicates per treatment, except for the Western blot analysis. Data were analyzed with analysis of variance (ANOVA). Waller-Duncan's multiple comparison test was used to locate differences, using Statistics Package for Social Science (SPSS). Data are shown as the means ± standard deviation (SD). A probability value (*p*) of less than 0.05 was considered statistically significant.

## Abbreviations

ART: advanced assisted reproductive technologies
BBB scale: Basso, Beattie, Bresnahan scale
DAB: diaminobenzidine
ER: endoplasmic reticulum
ES-BTB-hemidesmosome axis: ectoplasmic specialization–blood–testis barrier–hemidesmosome/ basement membrane functional axis
HMGB-1: high-mobility group box 1
HO-1: heme oxygenase-1
HS: hydrogen-rich saline
HRW: hydrogen-rich water
HRST group: hydrogen-rich saline-treated group
hSCI: hemi-sectioned spinal cord injury
ICSI: intracytoplasmic sperm injection
IVF: *in vitro* fertilization
MFN-2: mitofusin-2
MSET: mean seminiferous epithelial thickness
MSTD: mean seminiferous tubular diameter
POD: peroxidase
SCI: spinal cord injury
SD: standard deviation
TdT: terminal deoxynucleotidyl transferase
TEM: transmission electron microscopy
TUNEL: terminal deoxynucleotidyl transferase-mediated nick end labeling
